# Thermal and structural properties, and molecular dynamics in organic–inorganic hybrid perovskite (C_2_H_5_NH_3_)_2_ZnCl_4_

**DOI:** 10.1039/c9ra07695f

**Published:** 2019-11-21

**Authors:** Ae Ran Lim

**Affiliations:** Analytical Laboratory of Advanced Ferroelectric Crystals, Jeonju University Jeonju 55069 South Korea aeranlim@hanmail.net arlim@jj.ac.kr; Department of Science Education, Jeonju University Jeonju 55069 South Korea

## Abstract

The thermal and structural properties and molecular dynamics of layered perovskite-type (C_2_H_5_NH_3_)_2_ZnCl_4_ are investigated by differential scanning calorimetry, thermogravimetric analysis, and magic angle spinning nuclear magnetic resonance spectroscopy. The thermal properties and phase transitions are studied. Additionally, the Bloembergen–Purcell–Pound (BPP) curves for the ^1^H spin–lattice relaxation time *T*_1ρ_ in the C_2_H_5_NH_3_ cation and for the ^13^C *T*_1ρ_ in C_2_H_5_ are shown to have minima as a function of inverse temperature. This observation implies that these curves represent the rotational motions of ^1^H and ^13^C in the C_2_H_5_NH_3_ cation. The activation energies for ^1^H and ^13^C in the C_2_H_5_NH_3_ cation are obtained; the molecular motion of ^1^H is enhanced at the C-end and N-end of the organic cation, and that at the carbons of the main chain is not as free as that for protons at the C-end and N-end.

## Abstract 2

The thermal and structural properties and molecular dynamics of layered perovskite-type (C_2_H_5_NH_3_)_2_ZnCl_4_ are investigated by DSC, TGA, and MAS NMR spectroscopy.

## Graphical abstract

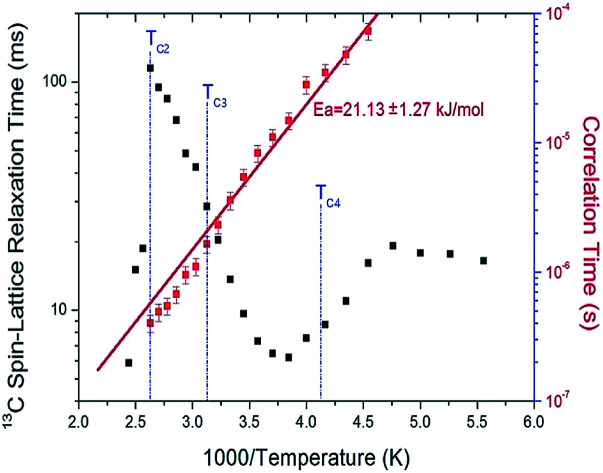

## I. Introduction

**Fig. 1:**
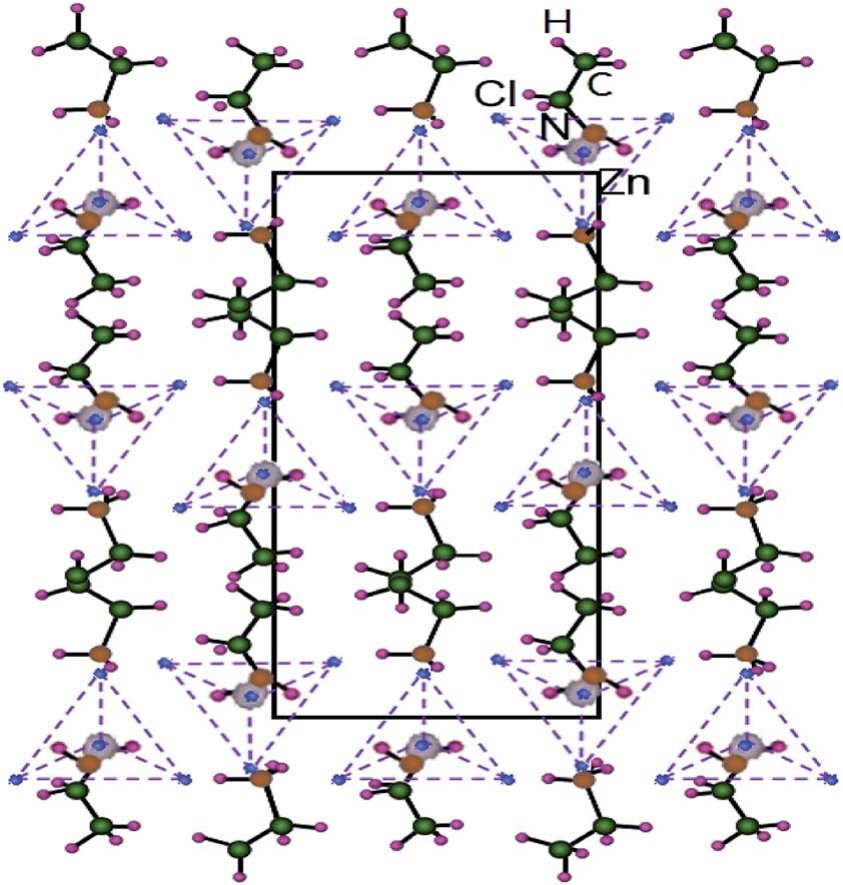
Orthorhombic structure of a (C_2_H_5_NH_3_)_2_ZnCl_4_ crystal for *bc*-plane at room temperature.^18^

Hybrid organic–inorganic compounds allow for the possibility of combining the properties of organic and inorganic materials at the molecular level.^1–4^ This class of hybrid materials is very broad and offers a wide set of different structures, properties, and potential applications.^5–12^ A new type of layered perovskite multiferroic, (C_2_H_5_NH_3_)_2_CuCl_4_, as a metal organic compound was found by Kundys *et al.*^6^ Multiferroics refer to materials that simultaneously have two or more of the following properties: spontaneous ferroelectricity, ferromagnetism, or ferroelasticity.^13^ (C_2_H_5_NH_3_)_2_CuCl_4_ crystallizes in a layered perovskite structure consisting of nearly isolated layers of corner-sharing ZnCl_6_ octahedra, and the interlayer distance is approximately 10 Å, where the layers are separated by two layers of ethylammonium cations (C_2_H_5_NH_3_)^+^.^14^ The NH_3_ polar heads of the chains are linked to the chlorine ions of the ZnCl_6_ octahedra by three hydrogen bonds N–H⋯Cl. The organic chains are joined by weak hydrogen bonds from the NH_3_ groups to the Cl ions. One interesting series is the metal–organic hybrids of chemical formula such as (C_2_H_5_NH_3_)_2_ZnCl_4_ with perovskite-type transition metal salts. A study of the electrical, dielectric, and optical properties of (C_2_H_5_NH_3_)_2_ZnCl_4_ was reported by Mohamed *et al.*^15^ They found that (C_2_H_5_NH_3_)_2_ZnCl_4_ is a layered perovskite-type compound that undergoes five phase transitions at 231 K, 234 K, 237 K, 247 K, and 312 K as determined by differential scanning calorimetry (DSC).^15^ The intensities of the endotherm peaks at 231 K, 237 K, and 312 K are very weak and perhaps correspond to second-order transformations. Tello^16^ reported a ferroelastoelectric phase transition at 243.3 K by optical and X-ray measurements with a group theoretical analysis. The phase transitions in this crystal are mostly connected to changes in the arrangement of the alkylammonium chains. The crystal structure of (C_2_H_5_NH_3_)_2_ZnCl_4_ at room temperature is orthorhombic. Fig. 1 reveals that its atomic arrangement can be described by an alternation of the organic and inorganic entities in the *bc* plane. This compound is characterized by two simple hydrogen bonds N–H⋯Cl linking the organic (C_2_H_5_NH_3_)^+^ cation to the (ZnCl_4_)^2−^ tetrahedral anions.^17^ This compound is crystallized in the orthorhombic system with a space group of *Pna*2_1_ at room temperature, and the lattice parameters are *a* = 10.043 Å, *b* = 17.594 Å, *c* = 7.397 Å, and molecules per unit cell, *Z* = 4.^18^ The Zn atoms in (C_2_H_5_NH_3_)_2_ZnCl_4_ are tetrahedrally coordinated, while the Cu atoms in (C_2_H_5_NH_3_)_2_CuCl_4_ are octahedrally coordinated. Although the chemical composition of the Zn and Cu compounds is similar, the coordination of the metal atom is different.

The structural geometry and molecular dynamics of the organic molecules within the layered hybrid structure are important for determining the influence of temperature on the evolution of structural phase transitions in the perovskite structure. Physical properties in particular depend on the characteristics of the metallic anion and the organic cation. Until now, the physical properties of (C_2_H_5_NH_3_)_2_ZnCl_4_ have been reported by a few research groups, whereas the thermal properties and molecular dynamics have not been studied.

The goal of this work is to analyze the crystal growth, thermodynamic properties, and structural dynamics of a (C_2_H_5_NH_3_)_2_ZnCl_4_ single crystal. The analysis is based on DSC, thermogravimetric analysis (TGA), and magic angle spinning nuclear magnetic resonance (MAS NMR). We measured the line widths, chemical shifts, and spin–lattice relaxation times (*T*_1ρ_) in the rotating frame using ^1^H MAS NMR, ^13^C cross-polarization (CP)/MAS NMR, and ^14^N static NMR as a function of temperature. The molecular dynamics of the (C_2_H_5_NH_3_) cation were investigated near the phase transition temperatures, and we discussed the activation energies for the molecular dynamics of C_2_H_5_ and NH_3_ in the (C_2_H_5_NH_3_)^+^ cation. Furthermore, we have compared the molecular motions of (C_2_H_5_NH_3_)_2_ZnCl_4_ obtained here and those of the previously reported (C_2_H_5_NH_3_)_2_CuCl_4_.

## II. Experimental methods

Crystals of (C_2_H_5_NH_3_)_2_ZnCl_4_ were prepared by dissolving stoichiometric amounts of the starting materials, commercial CH_3_CH_2_NH_2_·HCl (ethylamine hydrochloride, Aldrich 98%) and ZnCl_2_, in water. Single crystals were grown by a slow evaporation of the aqueous solution at room temperature. The obtained crystals were colorless and hexagonal in shape.

The thermal stability was checked by means of TGA and optical polarizing microscopy. The TGA curve at a heating rate of 10 °C min^−1^ was measured under N_2_ atmosphere, and the mass of the powdered sample used in the TGA experiment was 6.63 mg. The phase transitions were performed by DSC in the temperature range of 300–670 K with 10 °C min^−1^ heating rates.

The line widths, chemical shifts, and *T*_1ρ_ values for (C_2_H_5_NH_3_)_2_ZnCl_4_ were obtained by ^1^H MAS NMR and ^13^C CP/MAS NMR at Larmor frequencies of *ω*_0_/2π = 400.13 and 100.62 MHz, respectively, using a Bruker 400 MHz NMR spectrometer at the Korea Basic Science Institute, Western Seoul Center. Powdered samples were placed within a 4 mm CP/MAS probe, and the MAS rate for ^1^H and ^13^C measurements, to minimize spinning sideband overlap, was set to 10 kHz. The ^1^H *T*_1ρ_ values were determined using a π/2 − *t* sequence by varying the duration of spin-locking pulses. ^13^C *T*_1ρ_ values were measured by varying the duration of the spin-locking pulse applied after the CP preparation period. The width of the π/2 pulse used for measuring *T*_1ρ_ for ^1^H and ^13^C was 3.7 μs, with the spin-locking field set at 67.56 kHz. The chemical shifts and *T*_1ρ_ were measured over a temperature range of 180–430 K.

In addition, the ^14^N NMR spectra of the (C_2_H_5_NH_3_)_2_ZnCl_4_ single crystals in the laboratory frame were measured using a Unity INOVA 600 NMR spectrometer at the same facility. The static magnetic field was 14.1 T and the Larmor frequency was set to *ω*_0_/2π = 43.345 MHz. The ^14^N NMR experiments were conducted using a solid-echo (π/2–*t*–π/2–*t*) pulse sequence. The width of the π/2 pulse was 4 μs.

## III. Experimental results

**Fig. 2:**
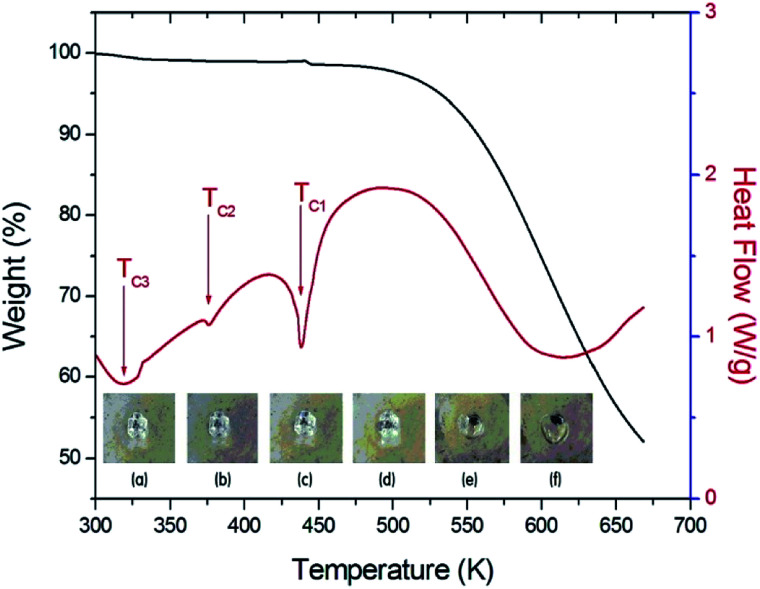
TGA and DSC curves of (C_2_H_5_NH_3_)_2_ZnCl_4_ crystal (inset: changes of a (C_2_H_5_NH_3_)_2_ZnCl_4_ crystal according to the temperature: (a) 300 K, (b) 400 K, (c) 450 K, (d) 460 K, (e) 470 K, and (f) 475 K).

Fig. 2 shows the simultaneous TGA and DSC curves for the (C_2_H_5_NH_3_)_2_ZnCl_4_ single crystal. A drastic weight loss onset occurred at 460 K (=*T*_d_), which is attributed to the beginning of the evaporation of C_2_H_5_NH_2_ and HCl due to partial thermal decomposition. The sample lost weight sharply between 500 K and 670 K, with a corresponding weight loss of 48%, which agrees almost exactly with the content of the organic component (45.53%) of C_2_H_5_NH_3_Cl in (C_2_H_5_NH_3_)_2_ZnCl_4_. DSC studies on the perovskite were undertaken to demonstrate structural transitions below the melting/decomposition point. In the DSC curve, the endotherm peaks at 320 K, 376 K, and 438 K correspond to the phase transitions. The (C_2_H_5_NH_3_)_2_ZnCl_4_ undergoes phase transitions at 438 K, 376 K, and 320 K, which are denoted as the *T*_C1_, *T*_C2_, and *T*_C3_ with decreasing temperature. As the temperature increases, the crystal is constantly colorless and transparent (300 K, 400 K, 450 K), and then it starts melting near 460 K. The crystal is melted near 475 K, as shown in the inset in Fig. 2.

**Fig. 3:**
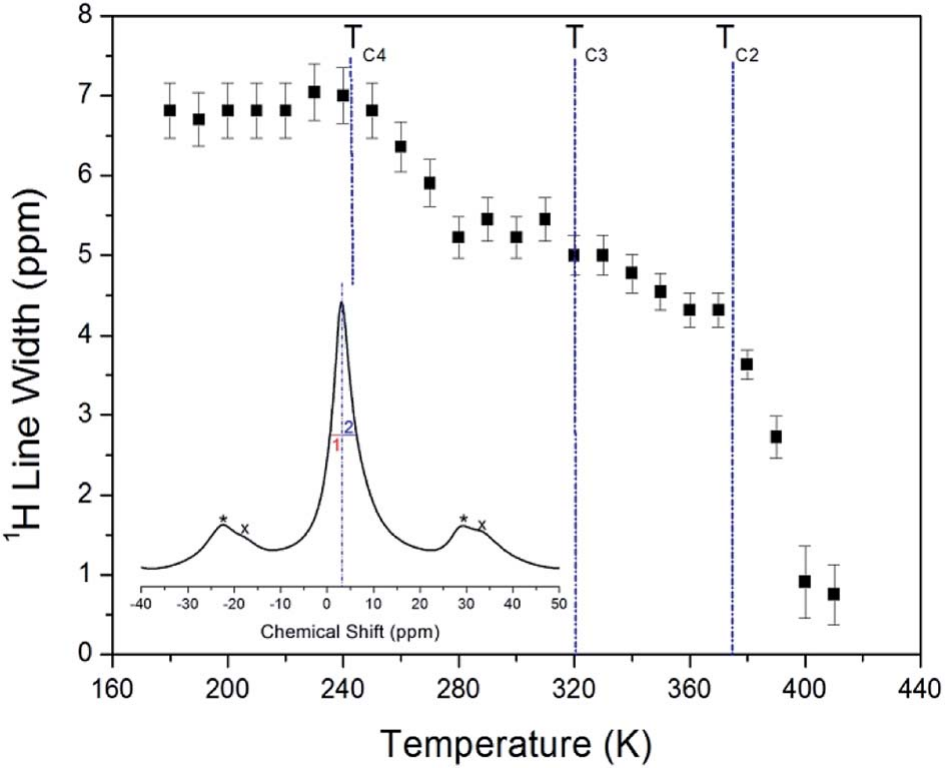
Line widths for ^1^H MAS NMR spectrum of (C_2_H_5_NH_3_)_2_ZnCl_4_ as a function of temperature (inset: ^1^H MAS NMR spectrum of (C_2_H_5_NH_3_)_2_ZnCl_4_ represented spinning sidebands indicated by asterisks and crosses at 300 K).

The ^1^H NMR spectra at a frequency of 400.13 MHz were obtained by MAS NMR. The ^1^H spectrum recorded at 300 K is shown in the inset in Fig. 3. The observed resonance line has an asymmetric shape, as the full-width at half-maximum (FWHM) values on the left side (symbol 1) and right side (symbol 2) are not the same. The asymmetric line shape is attributed to the two overlapping lines for C_2_H_5_ and NH_3_. Additionally, the FWHM line width narrows significantly with increasing temperature, as shown in Fig. 3. Note that there is no abrupt change in the line width near *T*_C3_ and *T*_C4_, whereas near *T*_C2_ it is abruptly decreased. Here, the *T*_C4_ (=247 K) is the previously reported phase transition temperature.^16^

**Fig. 4:**
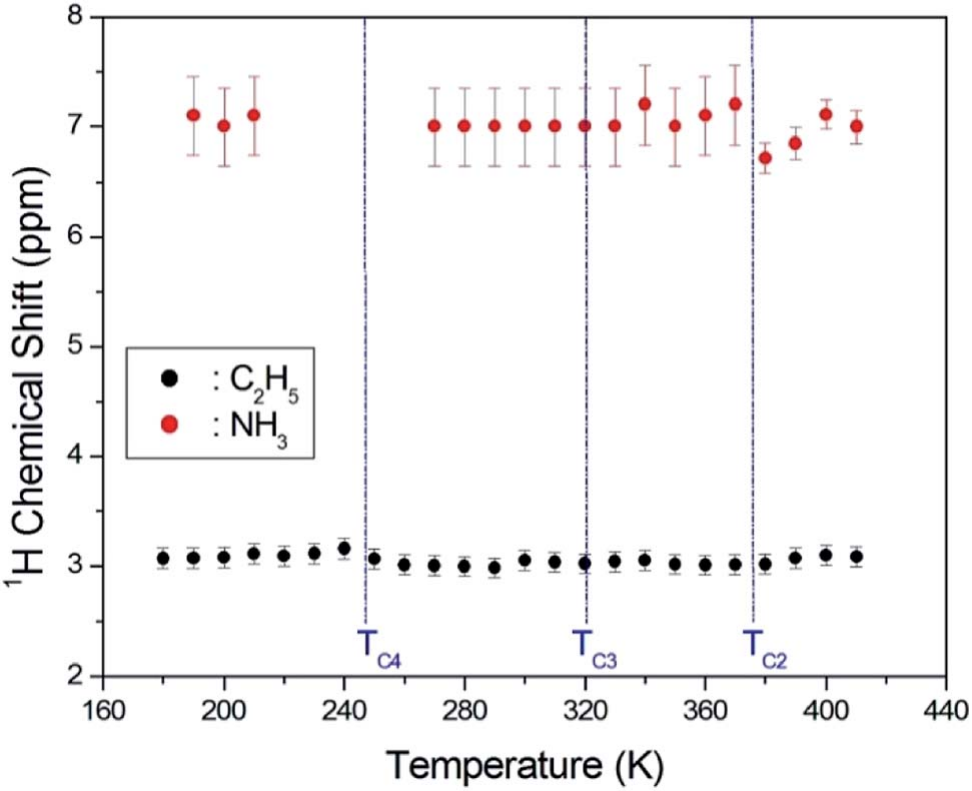
Chemical shifts for ^1^H MAS NMR of (C_2_H_5_NH_3_)_2_ZnCl_4_ as a function of temperature.

The asymmetric line shape in the inset in Fig. 3 is attributed to the C_2_H_5_ and NH_3_ overlapping lines. Thus, the overlapping peak is very broad. However, the spinning sidebands of the two types are evident in Fig. 3. Therefore, the peaks for C_2_H_5_ and NH_3_ by distance of sidebands can be distinguished. The spinning sidebands for C_2_H_5_ were marked with asterisks, while those for NH_3_ were marked with crosses. The peak of the lower chemical shift was attributed to ^1^H in C_2_H_5_, while that of the higher chemical shift was attributed to ^1^H in NH_3_. The spectrum at 300 K consisted of two peaks at chemical shifts of *δ* = 3.05 ppm and *δ* = 7 ppm, which were assigned to the ^1^H in the ethyl group C_2_H_5_ and the ammonium group NH_3_, respectively. The ^1^H chemical shifts for methyl and ammonium groups were nearly constant with temperature, as shown in Fig. 4.

**Fig. 5:**
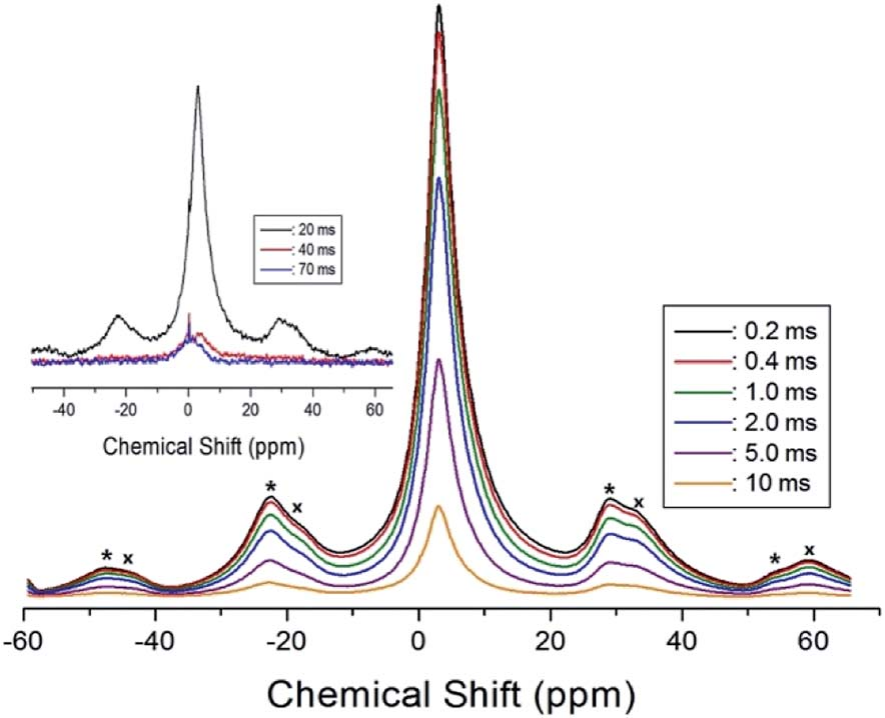
The ^1^H recovery traces as a function of the delay time at 300 K.

In order to obtain the ^1^H relaxation time values, the magnetization recovery curves as a function of delay time were measured at several temperatures for (C_2_H_5_NH_3_)_2_ZnCl_4_. The magnetization recovery curves for various delay times for ^1^H at 300 K are shown in Fig. 5. All traces obtained can be described by the single-exponential function^19,20^

**Fig. 6:**
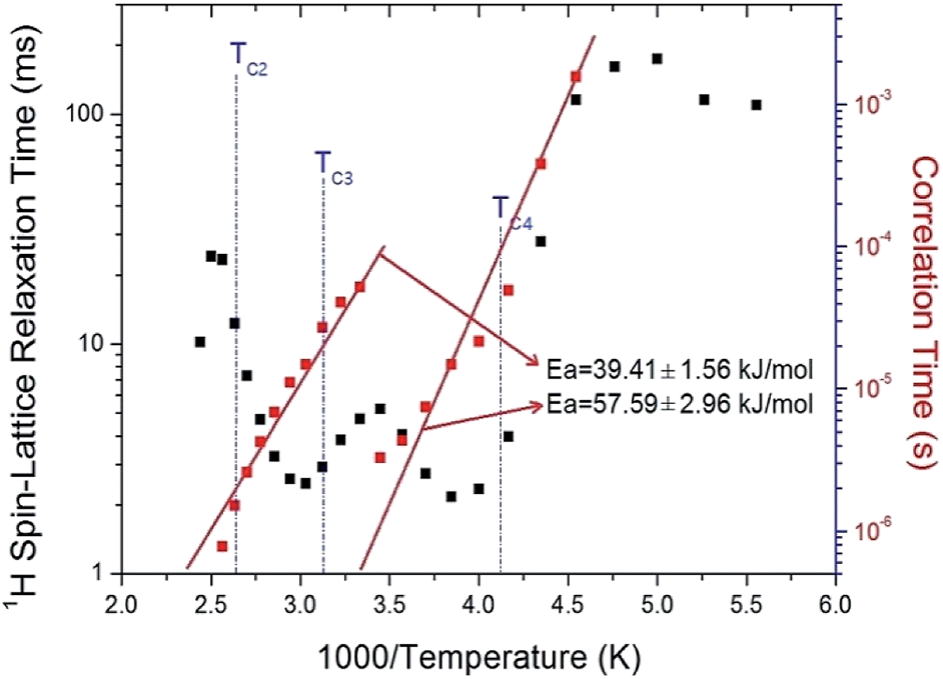
^1^H spin–lattice relaxation times *T*_1ρ_ in the rotating frame and correlation time of (C_2_H_5_NH_3_)_2_ZnCl_4_ as a function of inverse temperature.

1 *P*(*t*)/*P*_0_ = exp(−*t*/*T*_1ρ_),

where *P*(*t*) is the magnetization as a function of the spin-locking pulse duration *t*, and *P*_0_ is the total nuclear magnetization of the proton at thermal equilibrium. The recovery traces are shown for delay times ranging from 0.2 ms to 70 ms. The *T*_1ρ_ values were obtained from the slopes of the delay time *vs.* intensity. This analysis method was used to obtain the *T*_1ρ_ values for the protons, which are plotted as a function of inverse temperature in Fig. 6. From these results, the spin–lattice relaxation time in the rotating frame was obtained, and its temperature dependences are shown in Fig. 6.

The *T*_1ρ_ values of protons in the (C_2_H_5_NH_3_)_2_ZnCl_4_ are almost continuous near *T*_C3_ and *T*_C4_, and these values are on the order of a few milliseconds. The *T*_1ρ_ values abruptly decreased with temperature in the region approaching *T*_C2_. The relaxation time for the ^1^H nucleus has minimum values of 2.17 ms and 2.48 ms at 260 K and 330 K, respectively. This feature of *T*_1ρ_ indicates distinct molecular motions. The *T*_1ρ_ values are related to the corresponding values of the rotational correlation time, *τ*_C_, which is a direct measure of the rate of molecular motion. For the spin–lattice relaxation time in the rotating frame, the experimental value of *T*_1ρ_ can be expressed in terms of the correlation time *τ*_C_ for the molecular motion as suggested by the Bloembergen–Purcell–Pound (BPP) theory:^21,22^

2 *T*_1ρ_^−1^ = (*N*/20)(*γ*_H_*γ*_C_*ħ*/*r*_H–C_^3^)^2^{4*f*(*ω*_1_) + *f*(*ω*_H_ − *ω*_C_) + 3*f*(*ω*_C_) + 6*f*(*ω*_H_ + *ω*_C_) + 6*f*(*ω*_H_)}; *f*(*ω*_1_) = *τ*_C_/(1 + *ω*_1_^2^*τ*_C_^2^), *f*(*ω*_H_ − *ω*_C_) = *τ*_C_/[1 + (*ω*_H_ − *ω*_C_)^2^*τ*_C_^2^], *f*(*ω*_C_) = *τ*_C_/(1 + *ω*_C_^2^*τ*_C_^2^), *f*(*ω*_H_ + *ω*_C_) = *τ*_C_/[1 + (*ω*_H_ + *ω*_C_)^2^*τ*_C_^2^], *f*(*ω*_H_) = *τ*_C_/(1 + *ω*_H_^2^*τ*_C_^2^).

Here, *γ*_H_ and *γ*_C_ are the gyromagnetic ratios for the ^1^H and ^13^C nuclei, respectively; *N* is the number of directly bound protons; *r*_H–C_ is the H–C internuclear distance; *ħ* is the reduced Planck constant; *ω*_H_ and *ω*_C_ are the Larmor frequencies of ^1^H and ^13^C, respectively; and *ω*_1_ is the frequency of the spin-locking field of 67.56 kHz. We analyzed our data assuming that *T*_1ρ_ would show a minimum when *ω*_1_*τ*_C_ = 1, and that the BPP relation between *T*_1ρ_ and the characteristic frequency *ω*_1_ could be applied. We sensitively controlled the minima in the *T*_1ρ_ temperature variations and the slopes around the minima. From these results, the value of (*γ*_H_*γ*_C_*ħ*/*r*_H–C_^3^)^2^ for the constant in eqn (2) was obtained. We then calculated the temperature dependences of the *τ*_C_ values for protons by using the obtained values of (*γ*_H_*γ*_C_*ħ*/*r*_H–C_^3^)^2^. The temperature dependence of *τ*_C_ follows a simple Arrhenius equation:^21^

3 *τ*_C_ = *τ*_0_ exp(−*E*_a_/*RT*),

where *τ*_0_ is a pre-exponential factor, *T* is the temperature, *R* is the gas constant, and *E*_a_ is the activation energy. Thus, the slope of the linear portion of a semi-log plot should yield *E*_a_. The *E*_a_ value for the rotational motion can be obtained from the log *τ*_C_*vs.* 1000/*T* curve shown in Fig. 6; we obtained *E*_a_ = 39.41 ± 1.56 kJ mol^−1^ and *E*_a_ = 57.59 ± 2.96 kJ mol^−1^ for high and low temperatures, respectively. Here, *T*_1ρ_ and *E*_a_ for ^1^H are averaged for all hydrogens in the (C_2_H_5_NH_3_) cation. The rotational motion for protons at the end of the organic cation is more activated at the low temperature than at the high temperature.

**Fig. 7:**
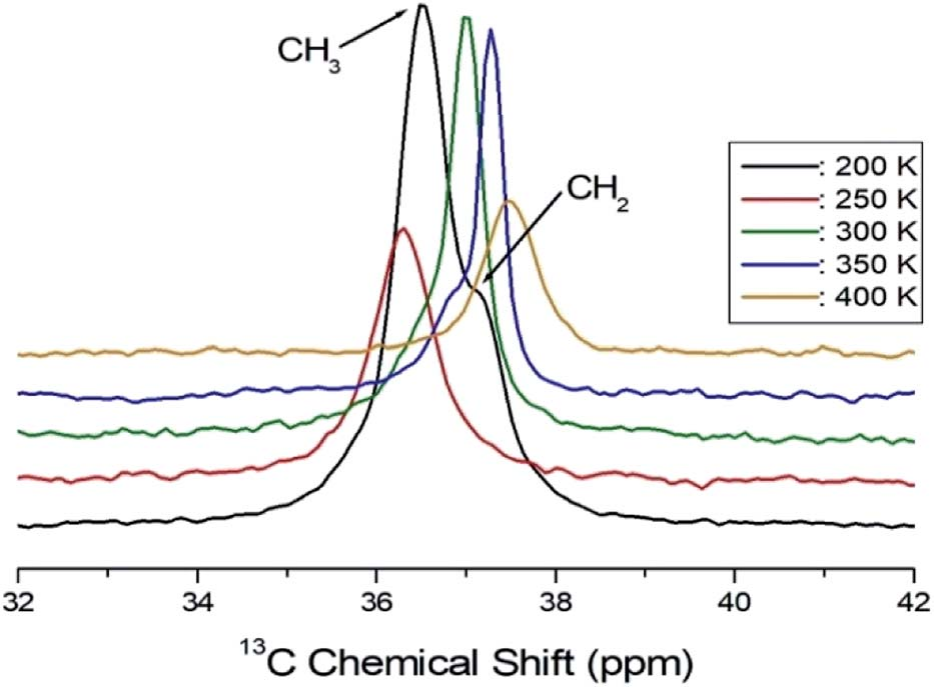
^13^C CP/MAS NMR spectra of (C_2_H_5_NH_3_)_2_ZnCl_4_ measured at different several temperatures.

Structural analysis of the ^13^C in C_2_H_5_ was also performed using ^13^C CP/MAS NMR. The ^13^C MAS NMR spectrum for (C_2_H_5_NH_3_)_2_ZnCl_4_ is shown in Fig. 7 as a function of temperature. The overlapped two signals in the spectrum for CH_3_ and CH_2_ in C_2_H_5_ are shown in Fig. 7. The resonance line has an asymmetric shape, similar to the ^1^H line shape. At 200 K, the chemical shifts of *δ* = 36.51 ppm and *δ* = 37.12 ppm with respect to tetramethysilane (TMS) are assigned to CH_3_ and CH_2_, respectively. The chemical shifts above 250 K were only continuous changes, whereas there was an abrupt change near 250 K. The change in the chemical shift is associated with a structural phase transition occurring at this temperature.

**Fig. 8:**
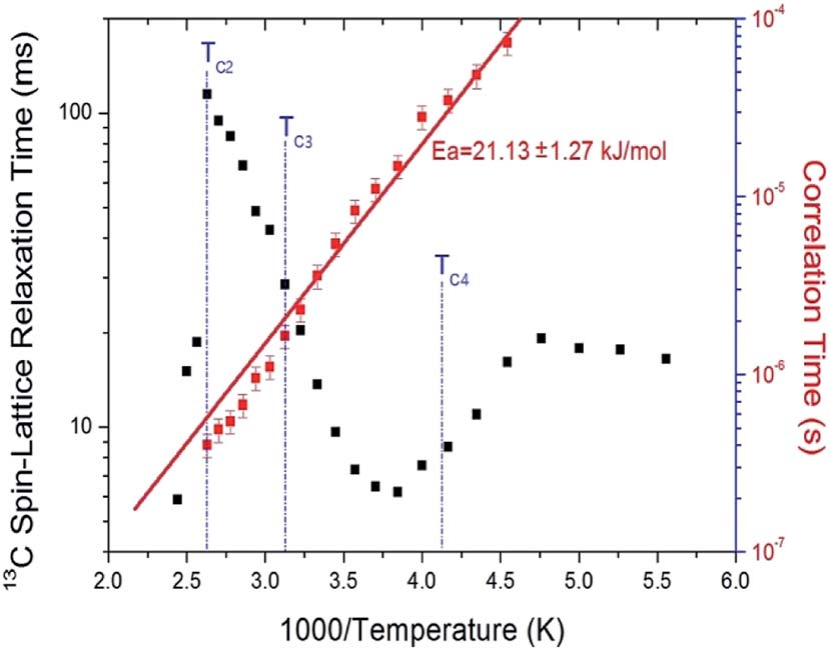
^13^C spin–lattice relaxation times *T*_1ρ_ in the rotating frame and correlation time of (C_2_H_5_NH_3_)_2_ZnCl_4_ as a function of inverse temperature.

The nuclear magnetization was also measured as a function of delay time in order to obtain the ^13^C *T*_1ρ_ values. The signal intensity of the nuclear magnetization recovery curves for ^13^C is described by a single exponential function of eqn (1) at all temperatures. The ^13^C *T*_1ρ_ values for C_2_H_5_ in (C_2_H_5_NH_3_)_2_ZnCl_4_ are plotted as a function of inverse temperature in Fig. 8. The ^13^C *T*_1ρ_ values near the phase-transition temperatures *T*_C3_ and *T*_C4_ are approximately continuous, whereas the *T*_1ρ_ near *T*_C2_ is abruptly decreased, similar to the ^1^H *T*_1ρ_. The *T*_1ρ_ value for carbon at room temperature is 13.65 ms. The *T*_1ρ_ curve below *T*_C2_ can be reproduced by BPP theory, and the BPP curve shows a minimum of 6.30 ms at 260 K. The correlation time for the rotational motion of C_2_H_5_ is obtained, and the activation energy from the log *τ*_C_*vs.* 1000/*T* curve shown in Fig. 8; we obtained *E*_a_ = 21.13 ± 1.27 kJ mol^−1^.

**Fig. 9:**
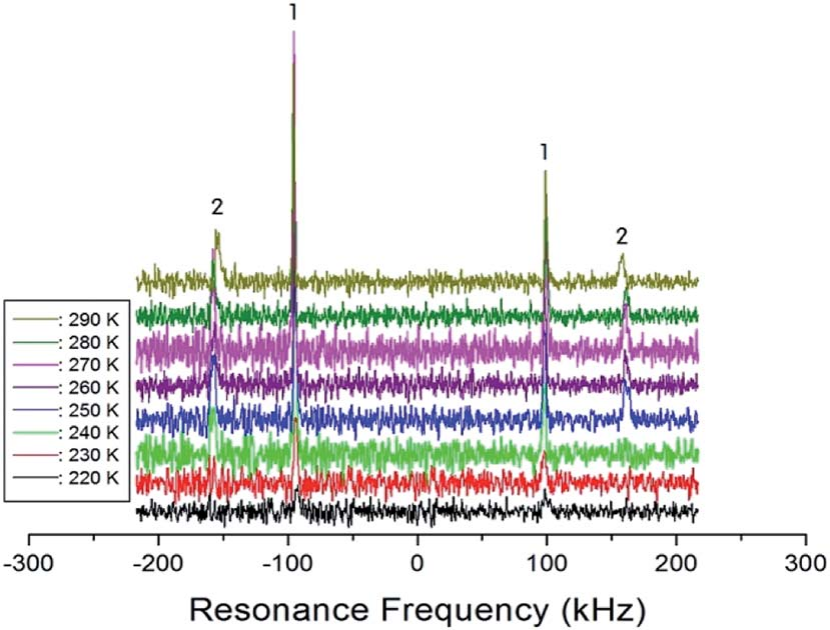
The resonance frequency of ^14^N NMR spectra in (C_2_H_5_NH_3_)_2_ZnCl_4_ single crystal between 220 K and 290 K.

In order to obtain information concerning the phase transition near 247 K (=*T*_C4_), the NMR spectrum of ^14^N (*I* = 1) in the laboratory frame was obtained. Two resonance signals with respect to NH_4_Cl were expected from the quadrupole interactions of the ^14^N nucleus. The ^14^N NMR spectra in (C_2_H_5_NH_3_)_2_ZnCl_4_ single crystals between 220 K and 290 K are plotted in Fig. 9. The number of resonance lines varies near 243 K; the ^14^N signals below 240 K show two resonance lines denoted by symbol 1, whereas those above 240 K show four resonance lines denoted by symbols 1 and 2. These four signals are attributed to the N(1) and N(2) sites in the physically inequivalent NH_3_ (1) and NH_3_ (2) ions, respectively. The abrupt splitting of the ^14^N NMR line is related to the phase transition at 247 K. This splitting of the ^14^N resonance signals is nearly constant with temperature. However, the ^14^N NMR spectrum above 300 K could not be detected due to a low intensity.

## IV. Conclusion

We discussed the molecular motions of cations of Zn-based hybrid materials based on NMR studies. The present work is devoted to the crystal growth, DSC, TGA, and NMR spectroscopy of the (C_2_H_5_NH_3_)_2_ZnCl_4_ compound. The thermal stability at different temperatures was considered. The cation dynamics in a layered perovskite-type (C_2_H_5_NH_3_)_2_ZnCl_4_ single crystal were investigated as a function of temperature by ^1^H MAS NMR, ^13^C CP/MAS NMR, and ^14^N static NMR experiments. There was no jump in *T*_1ρ_ across the phase transition at *T*_C3_ and *T*_C4_, while *T*_1ρ_ showed a slight jump at *T*_C2_. To obtain detailed information about the cation dynamics of this crystal, the spin–lattice relaxation time *T*_1ρ_ in the rotating frame for both ^1^H and ^13^C were obtained, revealing that these atoms undergo rotational motions. The BPP curves for the ^1^H *T*_1ρ_ in C_2_H_5_NH_3_ cation and for the ^13^C *T*_1ρ_ in C_2_H_5_ were shown to have minima as a function of inverse temperature. This implies that these curves represent the rotational motions of ^1^H and ^13^C. The activation energy for ^1^H in the C_2_H_5_NH_3_ cation is *E*_a_ = 39.41 kJ mol^−1^ above 290 K and 57.59 kJ mol^−1^ below 290 K, whereas that for ^13^C in the C_2_H_5_NH_3_ cation is 21.13 kJ mol^−1^. Furthermore, the carbon dynamics of C_2_H_5_ undergo rotation slower than protons. This implies that molecular motion is enhanced at the carbon-end and nitrogen-end of the organic cation, whereas molecular motion is not free at the main chain carbons of the organic cation.

Moreover, we compared the phase transition temperatures and molecular motions of the previously reported (C_2_H_5_NH_3_)_2_CuCl_4_ (ref. 23) and those of (C_2_H_5_NH_3_)_2_ZnCl_4_ studied here. The difference between these compounds is only the inorganic cation. (C_2_H_5_NH_3_)_2_ZnCl_4_ and (C_2_H_5_NH_3_)_2_CuCl_4_ are characterized by five (231, 234, 237, 247, and 312 K) and four (236, 330, 357, and 371 K) phase transitions, respectively. Furthermore, the molecular motions affecting the spin–lattice relaxation time *T*_1ρ_ in (C_2_H_5_NH_3_)_2_ZnCl_4_ are very different from those for (C_2_H_5_NH_3_)_2_CuCl_4_. The activation energies obtained from *T*_1ρ_ by the ^1^H and ^13^C measurements for the two compounds are summarized in Table 1. The *E*_a_ for ^1^H in (C_2_H_5_NH_3_)_2_ZnCl_4_ are the values at high temperatures above 290 K and the low temperatures below 290 K, respectively. In the case of (C_2_H_5_NH_3_)_2_CuCl_4_, *E*_a_ for each C_2_H_5_ and NH_3_ is shown at a temperature range from 180 K to 240 K.^23^ The values of *E*_a_ obtained from the ^1^H measurements of (C_2_H_5_NH_3_)_2_ZnCl_4_ are larger than those of (C_2_H_5_NH_3_)_2_CuCl_4_, whereas those obtained from the ^13^C measurments are similar. These results indicate that the activation energies obtained from the ^1^H measurements for the H–Cl–Zn bond in (C_2_H_5_NH_3_)_2_ZnCl_4_ without the paramagnetic ions is larger than that for the H–Cl–Cu bond in (C_2_H_5_NH_3_)_2_CuCl_4_ including paramagnetic ions. These differences are due to the differences in the electronic structure of the Zn^2+^ and Cu^2+^ ions, particularly, the d electrons, which screen the nuclear charge from the motion of the outer electrons. Zn^2+^ has filled d shell, whereas Cu^2+^ has one s electron outside the closed d shell. It is also likely due to several other factors, such as different coordination of the metal atom, different lattice constants, and hydrogen bonding strength. This suggests that the differences in the chemical properties of metal ions are responsible for the variations in the characteristics of the phase transitions and molecular motions in these crystals. This study can motivate us to find a solution for improving the material features as well as solar cell performance using lead-free perovskites based on Zn or Cu in market-competitive optoelectronic materials for photovoltaics (PV) and light emitting diodes (LED) applications.^3,24,25^

**Table 1:** Activation energies, *E*_a_ (kJ mol^−1^) for ^1^H and ^13^C nuclei in (C_2_H_5_NH_3_)_2_ZnCl_4_ and (C_2_H_5_NH_3_)_2_CuCl_4_

	(C_2_H_5_NH_3_)_2_ZnCl_4_	(C_2_H_5_NH_3_)_2_CuCl_4_ (ref. 23)
^1^H	39.41 ± 1.56 (for all hydrogen above 290 K)	12.19 ± 1.30 (for C_2_H_5_ below 240 K)
^1^H	57.59 ± 2.96 (for all hydrogen below 290 K)	8.33 ± 0.50 (for NH_3_ below 240 K)
^13^C	21.13 ± 1.27 (for C_2_H_5_)	21.35 ± 0.45 (for CH_3_)
^13^C		19.72 ± 1.76 (for CH_2_)

## Conflicts of interest

There are no conflicts to declare.

## Supplementary Material
